# Frequency of Abruptio Placenta in Women With Pregnancy-Induced Hypertension

**DOI:** 10.7759/cureus.21524

**Published:** 2022-01-23

**Authors:** Saba Khan, Geeta Chughani, Farheen Amir, Khadija Bano

**Affiliations:** 1 Department of Obstetrics and Gynecology, Jinnah Postgraduate Medical Centre, Karachi, PAK

**Keywords:** pre-eclampsia, morbidity and mortality, frequency, pregnancy induced hypertension, abruptio placenta

## Abstract

Background: Bleeding that takes place after premature separation of the normally situated placenta, usually after 20 weeks of pregnancy, is known as abruptio placenta. Factors increasing chances of abruptio placenta are advanced age pregnancy, parity, smoking, pregnancy-induced hypertension, pre-eclampsia, and previous incidence of abruptio. The objective of the study was to find the frequency of abruptio placenta in women with pregnancy-induced hypertension.

Methods: This descriptive prospective study was conducted in the Department of Obstetrics and Gynaecology, Jinnah Postgraduate Medical Centre, Karachi, from January to July, 2021. Women of gestational age above 20 weeks were included. Patients with blood pressure ≥140/90mmHg were considered as having pregnancy-induced hypertension. Early separation of the normally placed placental from the uterine wall was defined as placental abruption with clinical signs of painful vaginal bleeding (concealed or revealed), uterine contractions, and non-reassuring fetal heart rate. Descriptive statistics were calculated. Stratification was done and the post-stratification chi-square test was applied. P-value ≤ 0.05 was taken as significant.

Results: A total of 205 patients were included in the study. The mean age was 24.26±2.92 years. The mean gestational age was 30.82±3.22 weeks. The mean parity was 2.59±0.80 children. Mean systolic blood pressure was 148.48±5.99 mmHg and mean diastolic blood pressure was 94.85±3.05 mmHg. Bleeding was reported in 110 (53.7%) cases. Lower abdominal tenderness in 125 (60.5%) cases. Fetal heart rate was normal in 16.6% of the cases. Abruptio placenta was observed in 29 (14.1%) patients.

Conclusion:Abruptio placenta is a life-threatening condition that occurs during pregnancy that can result in both maternal and fetal morbidity and mortality. Adequate and urgent intervention can result in a favourable outcome.

## Introduction

Pregnancy-induced hypertension (PIH) is the onset of new arterial hypertension in a pregnant woman after the 20th week of gestation, during labour or puerperium in previously normotensive women without the presence of protein in the urine [1,2]. It occurs in about 6-8% of all pregnancies [1,3]. The incidence of PIH varies throughout the world; however, the incidence is higher in developing countries [4]. The risk of PIH is estimated at 1:1500 pregnancies and is responsible for a high number of maternal and fetal deaths worldwide [5]. The major complications include eclampsia, abruptio placenta (AP), intrauterine growth retardation (IUGR), low birth weight (LBW) babies, fetal distress, and neonatal deaths [6].

If a normally planted placenta separates from the uterus after 20 weeks of pregnancy with either visible or hidden bleeding per-vaginum, the condition is defined as AP [7]. Painful bleeding per-vaginum may be associated with a tense tender abdomen due to concealed blood. This may also be an addition to painful uterine contractions and non-reassuring cardiotocograph (CTG) [8].

Worldwide occurrence of premature separation of the placenta is seen in nearly 1% of all pregnancies [9]. Associated risk factors with placental abruption are advanced age of mother, smoking both in father and mother, previous history of abruption, increased parity, twin pregnancy, as well as conditions like polyhydramnios, chorioamnionitis, clotting disorders, and trauma to the abdomen [10]. Studies specific to placental abruption in PIH are still lacking. In a study conducted in Albania in 2013, various complications were reported in PIH, which included renal impairment (12.3%), AP (7.0%), eclampsia (3.3%) and disseminated intravascular coagulation (2.8%) [11]. 

The prevalence of PIH is still high worldwide. Research has been done internationally regarding risk factors, complications, and outcomes of PIH in association with preeclampsia and eclampsia. In contrast, local literature on this subject is still devoid of much-needed data. The present study is designed to focus on collecting local data on this subject and to objectively determine its impact on the outcome of pregnancy complicated with PIH in terms of improving fetal and maternal morbidity and mortality

## Materials and methods

This descriptive cross-sectional study was conducted at the Department of Obstetrics and Gynaecology, Jinnah Postgraduate Medical Centre, Karachi, for a period of six months from January 25 to July 24, 2021, after approval of the Institutional Review Board committee (F.2-81/2021-GENL/73089/JPMC).

Sample size and technique

The sample size of the study was calculated by using the WHO sample size calculator taking confidence interval = 95%. Anticipated population proportion (prevalence of AP in PIH patients) =7%. Absolute precision required = 3.5%. The sample size turned out to be 205 patients with PIH. Non-probability consecutive sampling was used for the study.

Sample selection

Inclusion criteria included: (1) gestational age 20 weeks onwards assessed on ultrasound pelvis, (2) singleton pregnancy on dating ultrasound, (3) blood pressure ≥140/90 in absence of proteinuria on two separate occasions of at least six hours apart.

Exclusion criteria included: (1) gestational age below 20 weeks assessed on ultrasound pelvis, (2) multifetal pregnancy, (3) women with a history of smoking, (4) medical disorders like diabetes mellitus (FBS > 126mg/dl), heart disease (EF < 25%), and renal disease (serum Cr > 3 mg/dl), (5) history of trauma in the current pregnancy, and (6) pre-eclampsia confirmed on urinary protein > 3g/dl.

Data collection procedure

With the consent of the hospital ethical committee, data was collected for this cross-sectional study. Consent was taken from women who satisfied the inclusion criteria and were included in the study by non-probability consecutive sampling method. Confidentiality of data was ensured.

Data analysis procedure

Data were entered and analyzed with the help of SPSS Statistics for Windows, Version 17.0 (released 2008, SPSS Inc., Chicago, United States). Mean and standard deviation was computed for quantitative variables like maternal age, gestational age, and blood pressure. Frequency and percentage were presented for qualitative variables like parity (nulliparous, multiparous) and the outcome variable, i.e. abruptio placenta. Stratification of maternal age, gestational age, and parity (nulliparous, multiparous) were done to see their effect on outcome variables by applying the chi-square test. P-value < 0.05 was taken as significant.

Operational definition

Pregnancy Induced Hypertension (PIH)

It was defined as blood pressure ≥ 140/90 recorded on two separate occasions six hours apart, in absence of proteinuria, arising after the 20th week of gestation in a previously normotensive woman.

Abruptio Placenta (AP)

It was defined as the early separation of the normally placed placental from the uterine wall. Clinical presentation of patients with abruptio placentae typically is painful vaginal bleeding. Bleeding was labeled positive if there were more than two wet pads in a day. It was diagnosed on clinical features and the identification of retroplacental clots on ultrasound.

## Results

A total of 205 females were included in this study. The overall mean age of study subjects was 24.26±2.92 years, mean gestational age was 30.82±3.22 weeks, and mean parity was 2.59±0.80. The overall mean systolic blood pressure was 148.48±5.99 mmHg and mean diastolic blood pressure was 94.85±3.05 mmHg. Among the 205 subjects, bleeding was reported in 110 (53.7%) cases and tenderness in 124 (60.5%) cases while fetal heart rate was normal in 16.6% of study subjects. In our study, 29 (14.1%) patients were found with AP. Fetal mortality was seen in 32 (15.6%), 18 (56.25%) of which were seen in AP patients. Table 1 shows detailed stratification and characteristics of the patients.

**Table 1:** Characteristics of the study participants

Age		
Mean	24.26±2.92	
<=25 years	128	62.4%
>25 years	77	37.6%
Gestational age		
Mean	30.82±3.22	
<=30 weeks	90	43.9%
>30 weeks	114	55.6%
Parity		
Mean	2.59±0.80	
Nulliparous	15	7.3%
Multiparous	190	92.7%
Blood pressure		
Systolic Blood Pressure (Mean)	148.48±5.99	
Diastolic Blood Pressure (Mean)	94.85±3.05	
Bleeding	110	53.7%
Tenderness	124	60.5%
Fetal heart rate		
Absent	154	75.1%
Normal	34	16.6%
Present	17	8.3%
Abruptio placenta		
	29	14.1%

The results showed that there was a significant association of AP with maternal age (p=0.011), gestational age (p=0.006), parity (p=0.010), and absent fetal heart rate (p=<0.001). Table 2 shows the stratification of AP patients.

**Table 2:** Stratified analysis of abruptio placenta patients

	Abruptio placenta		
	Yes	No	P-value
	n-29	n-176	
Age			
Mean	25.3±2.7	24.1±2.9	
<=25 years	12	16	0.011
>25 years	17	60	
Fetal heart rate	18	14	<0.001
Gestational age			
Mean	32.0±2.8	30.6±3.3	
<=30 weeks	6	85	0.006
>30 weeks	23	91	
Parity			
Mean	2.2±1.2	2.7±0.7	
Nulliparous	6	9	0.01
Multiparous	23	167	

## Discussion

Normal growth and development of the fetus are supported by a healthy placenta, a specialized organ of pregnancy. The highly dynamic triad of placenta, fetus, and mother plays an important role in providing a conducive atmosphere for fetal growth. As pregnancy progresses, placental calcification occurs as its normal aging process. Under pathological circumstances, placental maturation may cause restriction of fetal growth resulting in fetal death [11,12].

PIH results due to progressive pathological calcification of the placenta. This phenomenon eventually accounts for reduced placental blood flow culminating in fetal growth restriction, LBW with preterm delivery as its outcome [13]. Numerous risk factors are associated with AP. Among these PIH, chronic hypertension, anemia, ischemic heart disease, parity, smoking, and prolonged rupture of membranes are regarded as important risk factors for abruption [14,15]. The authors have focused to determine any positive association of PIH with AP among the local population in Sindh, Pakistan. AP affects up to 4.4% of all pregnancies according to local and regional literature, which is clearly high compared to the global frequency of 1% [16,17].

In the present study, AP complicated 14.1% (29) of pregnancies with PIH. Other authors have quoted a risk of 10-28% among hypertensive patients [18,19,20]. In one study, an incidence of 78.5% was noted [21]. Two studies from the Middle East have similarly identified high rates of AP in PIH [22,23]. Some authors have identified higher maternal age of more than 35 years as a significant cause of AP [24]. Among women with the advanced age of more than 40 years, the occurrence of AP was found to be 2.3 times more likely when compared with the age group of less than 35 years [25]. Among our patients, the mean age of patients with abruption was 24.26±2.92 years. In a study by Prichard et al, grand multiparity was associated with the risk of abruption, and its incidence was found higher among women on their third pregnancy [25].

Abruption due to gestational hypertension was seen in 10% and 21% of cases among the local and regional populations, respectively [1,7,9]. The risk of abruption placenta was also found to be 2.4 times among women with chronic hypertension and not surprisingly increased further in the presence of preeclampsia or fetal growth retardation [26,27]. This observation is further strengthened by results obtained by Zetterstron et al., who reported twice as many cases of AP (incidence 1.1) in the presence of chronic hypertension when compared to normotensive women (0.5) [28]. A similar observation was reported in a study that identified a high incidence of fetomaternal poor outcomes among women with a higher frequency of PIH or chronic hypertension [29]. Several other studies have also confirmed this association [30,31].

Uncontrolled or poorly controlled risk factors when present simultaneously in a pregnant woman greatly accelerated placental insufficiency leading to AP. In a population-based study from Israel, a comparison was drawn between pregnant women with or without AP. Among a total of 184111 deliveries, 1365 were found to be in women with abruption. Further inquiry found that hypertension was the second most frequent cause of abruption both among placental abruption cases. However, non-abruption cases were also found to be hypertensive (15.3% vs. 5.9%.) In addition to these findings, other poor fetal outcomes like LBW were strongly associated with vascular dysfunction due to PIH and resulted in AP [32].

A local study published in 2006 further supported the available evidence that hypertension, grand-multiparity, anemia, and preeclampsia play significant roles as risk factors for AP [33]. Kramer et al., on the other hand, disagreed with multiparity as a risk factor for AP. In their opinion, the higher frequency of AP may be seen due to different ethnicities [15].

AP is a devastating complication for the mother and her child. The prevalence of this diagnosis is higher among our local population. Despite these facts, few publications are available on this topic in the international literature from our part of the world. Till now no conclusive etiological reason has been found for a higher incidence of AP. Furthermore, the condition cannot be accurately predicted with high sensitivity and specificity. It is an observation that the incidence of AP in subsequent pregnancies rises with the risk of a higher incidence of poor feto-maternal outcome [34]. Once recognized, delivery should be scheduled when fetal lung maturity is obtained between 34 to 37 weeks for a desirable outcome [35].

The use of low molecular weight heparin has been found to improve pregnancy outcomes in women at risk of AP, irrespective of thrombophilia status [36]. There may be a role of heparin in improving pregnancy outcomes in diseases involving the uteroplacental interface [37]. However, this needs to be tested in large randomized trials.

Study limitations

The current study has a few limitations. First, this study was conducted in a single hospital so it may not represent the experience of most other hospitals across the city. Second, most patients recruited belonged to a low socioeconomic status with very similar risk profiles for AP. Finally, the study was based on a relatively small sample size with non-randomization of the sample, the odds ratio was not found, and the results obtained may not be generalized to larger population groups.

## Conclusions

Hypertensive disorders frequently develop during pregnancy. AP often results in an unfavorable outcome for the mother and fetus. Our study results showed that there is a significant association of AP with advanced maternal age, PIH, gestational age, and parity.
